# Prevalence and characterization of carbapenem-resistant gram-negative bacteria from poultry in Tamil Nadu, India

**DOI:** 10.1016/j.onehlt.2025.101192

**Published:** 2025-09-05

**Authors:** Seshan Sivasankar, Martin Peter Grobusch, Frieder Schaumburg, Sankarganesh Jeyaraj

**Affiliations:** a PSG Center for Molecular Medicine and Therapeutics, PSG Institute of Medical Sciences and Research, Coimbatore, Tamil Nadu, India; b Department of Genetics and Molecular Biology, PSG Center for Genetics and Molecular Biology, Coimbatore, Tamil Nadu, India; c Center of Tropical Medicine and Travel Medicine, Department of Infectious Diseases, Amsterdam University Medical Centers, Amsterdam, the Netherlands; d Centre de RecherchesMédicales de Lambaréné CERMEL, Hospital Albert Schweitzer, BP 242 Lambaréné, Gabon; e Institut für Tropenmedizin, Eberhard Karls Universität Tübingen and German Center forInfection Research (DZIF), Tübingen, Germany; f Masanga Medical Research Unit, Masanga, Sierra Leone; g Institute of Infectious Diseases and Molecular Medicine, University of Cape Town, Cape Town, South Africa; h Institute of Medical Microbiology, University Hospital Münster, Münster, Germany

**Keywords:** Poultry, Enterobacterales, Carbapenem resistance, *K. pneumoniae*-ST16, WGS

## Abstract

Recent emergence of carbapenem-resistant bacteria in poultry has raised concerns on the potential transmission to humans.

We aimed to investigate the molecular epidemiology, genotype and phenotype of the carbapenem-resistant gram-negative bacteria isolated from raw chicken meat samples from India and to evaluate resistance and virulence genes through whole genome sequencing (WGS).

Poultry samples (*n* = 250) from local vendors in Coimbatore, Tamil Nadu, India were enriched in selective McConkey broth before culturing on McConkey agar (both containing 4 μg/ml meropenem). The isolates were screened for carbapenem resistance genes and carbapenemase production by PCR and Modified Hodge test respectively. Selected isolates underwent WGS for detailed analysis of the bacterial genomes.

Carbapenem-resistant bacteria were identified in 36.8 % (92/250) of chicken meat samples, from which 347 isolates were selected for further testing. Common species were *Morganella morganii*, *Providencia alcalifaciens*, *Stenotrophomonas pavanii, Klebsiella aerogenes*, *Proteus mirabilis*, *Providencia huaxiensis*, *Proteus terrae*, *Stenotrophomonas maltophilia and Klebsiella pneumoniae.* The carbapenemase genotypes detected were *bla*_NDM-1_14.7 % (51/347), *bla*_KPC-3_ 5.4 % (19/347) and *bla*_OXA-48_-like 7.2 % (25/347). Two isolates were found to co-produce *bla*_NDM-1_ and *bla*_OXA-48_-like*,* while three isolates co-produced *bla*_NDM-1_ and *bla*_KPC-3_*.*The majority of the PCR positive isolates were confirmed to produce carbapenemase in the Hodge test (*n* = 54/95). Two *K. pneumoniae* isolates (ST15 and ST16) carried resistance genes including *bla*_NDM-1_*, bla*_CTX-M_*, bla*_SHV_ and *bla*_OXA-232_; virulence factors id*ent*ified were *fep, ent, fyu, ybt*, *irp and OmpA,* alongside *yag/ ecp* and *ykg/ecp* complexes. This study highlights the need for the molecular surveillance studies in prominent poultry producing countries like India.

## Highlights

• 36.8 % of the chicken meat were contaminated
• Most common carbapenemases were *bla*_NDM-1_ (13.5 %), *bla*_KPC-3_ (5.1 %) and *bla*_OXA-48_-like (6.8 %).
• *K. pneumoniae* ST16 and ST15 carry *bla*_NDM-5_, *bla*_OXA-181_, *bla*_SHV-1_and *bla*_CTX-M-15_.

## 1 Introduction

Antimicrobial resistance (AMR) poses a universal threat to health care systems, which could result in ten million deaths annually by 2050 [1]. Amongst gram-negative bacteria, *Enterobacterales* exhibit resistance to a broad spectrum of antibiotics (e.g. Extended-spectrum beta-lactamase [ESBL]-producers), with high mortality rate [2]. Carbapenems are frequently used for treating infections caused by ESBL-producing *Enterobacterales* or *Acinetobacter* spp. and fall under the WHO AWaRe‘Watch’ category (i.e. their use should be restricted) [3]. Carbapenem-resistant *Enterobacterales* (CRE) were first reported in1985 [4,5]. The World Health Organization (WHO)has classified CRE as critically prioritized pathogens since 2017 due to their significant global health impact, with the status reaffirmed in 2024 [6].

The primary mechanism of carbapenem resistance is mediated by hydrolysing enzymes, particularly carbapenemases. The most common carbapenemases are New Delhi metallo-beta-lactamase (*bla*_NDM-1_), *Klebsiella pneumoniae* carbapenemase (*bla*_KPC-3_) and oxacillinase (*bla*_OXA-48-_like) [7]. Poultry products, especially retail chicken meat, act as a significant source of antimicrobial-resistant pathogens, such as ESBL producing *Enterobacterales* [8,9].This is largely attributed to the extensive use of antibiotics in poultry farming for growth promotion and therapeutic purposes [10]. Notably, the spread of carbapenem resistance in livestock has been observed, even though carbapenems are not used in animal farming, eliminating the possibility of direct selective pressure [11]. A study demonstrated that *Salmonella Corvallis* with the *bla*_NDM-1_ carrying plasmid (*IncA/C*_*2*_) isolated from an infected chicken was able to conjugate the plasmid into *Escherichia coli* and *K. pneumoniae.* In an another study, it was showed that antibiotic residues could persist in the chicken farms even when they were no longer used [12]. An increase in the prevalence of carbapenem-resistant bacteria, including *Proteus mirabilis*, *E. coli*, *K. pneumoniae*, *Alcaligenes faecalis*, *Pseudomonas putida and some of the members of Morganellaceae* has been reported in broiler farms that harboured carbapenemase genes [13]. In 2021, carbapenamase (*bla*_NDM-1_) producing *P. mirabilis* was reported in broiler chickens in China [14]. India is the fifth-largest producer of poultry meat, contributing 4.9 million tons of chicken meat in 2022 [15]. Tamil Nadu is the largest poultry producing state in India, with Coimbatore being the second largest poultry producer in the state next to Namakkal. India also exports chicken products to several countries, including the UAE, Oman, the Netherlands and Japan [16]. Although several studies have addressed the prevalence of carbapenem-resistant bacteria in clinical samples from Tamil Nadu, there is a lack of data on food samples, particularly raw chicken meat, from the region. [17,18]. This prompted the current study to evaluate the prevalence and genotypes of carbapenem-resistant *Enterobacterales* and other gram-negative bacteria in poultry from Coimbatore, Tamil Nadu, India. Furthermore, molecular typing, identification of resistance and virulence genes were performed using WGS.

## 2 Materials and methods

### 2.1 Sample collection

**Fig. 1:**
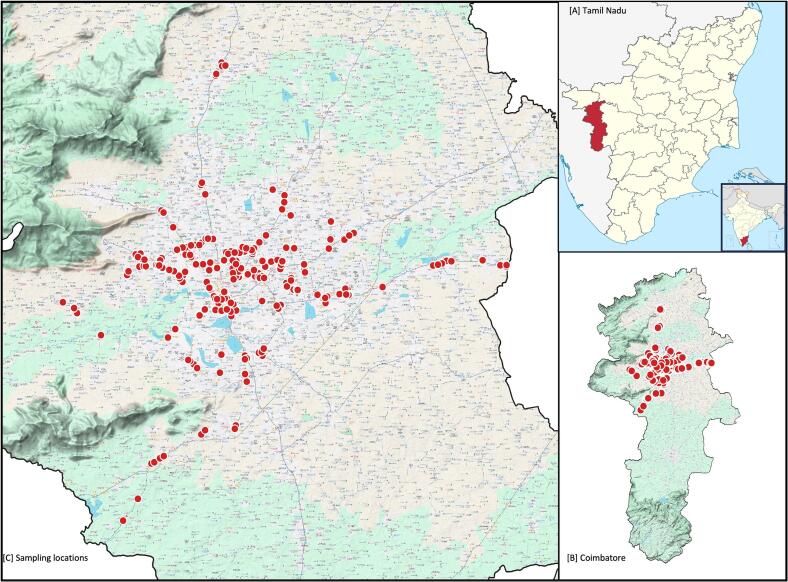
Chicken meat samples collection sites in Coimbatore, Tamil Nadu, India. A- Tamil Nadu state in India; B- boundary of Coimbatore; C- Sample locations in the Coimbatore. Image was created using Prizren QGIS software (v.3.34.2).

A total of 250 raw fresh chicken meat, each weighing approximately 30 g, were randomly collected from 250 different retail chicken meat vendors in Coimbatore, all supplied from local commercial farms. Not all the vendors from the region were subjected to sampling (for reasons such as the vendor was closed during the time of sampling, the vendor denied sampling, difficulty in reaching the location). Sampling was conducted in two phases from August 2019 to January 2020 (120 samples) and from December 2022 to January 2023 (130 samples). The geological coordinates of sampling locations are indicated in the Fig. 1.The sampling locations were marked and mapped using Prizren QGIS software (v.3.34.2). Each chicken meat sample was placed in a Whirl-Pak® closure bag (Wisconsin, USA) and labeled with relevant details, including the type of chicken meat, collection date and shop details. The sealed bags were transported to the laboratory in ice box to maintain sample integrity.

### 2.2 Bacterial isolation

Samples were subjected to bacterial isolation based on a previously published study, with slight modifications [19]. A total of 25 g of each sample was macerated aseptically using mortar and pestle, which were washed and sterilized between each sample. The sample was then enriched individually in 225 ml of peptone broth in Whirl-Pak closure bags (Wisconsin, USA) and incubated overnight at 37 °C with shaking at 180 rpm. After enrichment, the enriched peptone water (50 μl) was inoculated into five ml of MacConkey supplemented with a final concentration of 4 μg/ml meropenem for selective enrichment following CLSI guidelines (M100-33rd Edition). The selectively enriched broth was then spread-plated onto MacConkey agar supplemented with a final concentration of 4 μg/ml meropenem and incubated at 37 °C for 18–24 h. Following incubation, isolated colonies with distinct morphologies (size, shape, and colour) were further sub-cultured onto MacConkey agar supplemented with meropenem (4 μg/ml).

### 2.3 DNA extraction and bacterial identification

Using thermal lysis method, DNA extraction was carried out for all the isolates. Briefly, a loopful of bacterial colony was suspended in 100 μl of molecular-grade nuclease free water. The suspension was boiled at 100 °C for 10 min, followed by centrifugation at 3000 ×*g* for 10 min. The supernatant containing DNA was transferred to a fresh tube, and 140 μl of ice-cold absolute ethanol was added to precipitate the DNA. The precipitate was separated by centrifugation(20,000 ×*g* for20 minutes). The pellet was rinsed with 70 % ethanol and DNA was resuspended in TE buffer (10 mM Tris-HCl, 1 mM EDTA, pH 8.0). Species identification was performed using 16 s rRNA sequencing (Sanger)using primers 27F (5′-AGAGTTTGATCCTGGCTCAG-3′) and 1492R (5′-TACGGYTACCTTGTTACGACTT-3′) [21]. The amplicons were purified using the ExS-Pure™ Enzymatic PCR Purification Kit (Nimagen, Nijmegen, The Netherlands) according to the manufacturer's recommendations. Purified amplicons were subjected to sequencing using Genetic Analyzer 3500 (Applied Biosystems, Thermo Scientific, Hitachi Hi-tech, Japan), following the manufacturer's protocol. Raw sequence data and chromatogram files were visualized and analyzed in NCBI BLAST and EzBio Cloud for species identification. Query coverage of >99 % and species similarity score of >97 % were considered for the identification.

### 2.4 Minimum inhibitory concentrations

Minimum Inhibitory concentrations (MICs) of the isolates (*n* = 362) were determined for meropenem (Merck, Darmstadt, Germany) using the micro broth dilution method, and reported in accordance with CLSI guidelines (M100, 34th edition) [20]. All assays were conducted in duplicate using 96-well microtiter plates with cation-adjusted Mueller-Hinton broth (CaMHB). A 0.5 McFarland standard bacterial suspension was initially diluted (1:10) and meropenem was added to the wells at concentrations ranging from 128 μg/ml to 0.25 μg/ml. The first and last column served as the growth control and blank, respectively. The final parameters in each well were 5 × 10^5^ cells/ml, meropenem (128–0.25 μg/ml) and less than 1 % DMSO in a total volume of 100 μl. *E. coli* ATCC25922 was used as the as the control isolate. The plates were incubated at 37 °C for 18–20 h. The MIC was interpreted as the least concentration at which the growth was completely inhibited.

### 2.5 Modified Hodge test

The Modified Hodge Test (MHT) was used to evaluate the carbapenemase phenotype of all isolates. In this study, the MHT was performed using Triton X-100 as described previously [22]. Briefly, Triton X-100 (0.1 % *v*/v) was poured in the center of the cation-adjusted Muller-Hinton agar (caMHB) plate and evenly spread across the agar surface using a sterile swab until completely absorbed. *E. coli* ATCC 25922 was adjusted to the turbidity equivalent to the 0.5 McFarland standard and lawn cultured in the triton treated caMHB plates. A meropenem disc (10 μg) was placed in the center of the plate, and the test isolates were streaked as thin straight line from the disc in the center, towards the edge of the plate. The plates were rotated to 90^o^ degrees and second isolate was streaked. A maximum of four isolates per plate was streaked per plate. The plates were incubated at 37 °C for 18–24 h. Following incubation, results were interpreted as follows: test isolates that facilitated the growth of the *E. coli* ATCC 25922 along the periphery of the test isolate were considered as positive for carbapenemase production. This indicates the production of hydrolyzing enzymes capable for degrading the meropenem diffused in the agar.

### 2.6 Screening of carbapenemase genes

All isolates obtained from the selective enrichment, were screened for the five most common carbapenemase genes, including *bla*_NDM-1_*, bla*_KPC-3_*, bla*_OXA-48_-like*, bla*_VIM_ and *bla*_IMP_ by PCR and agarose gel electrophoresis (Table 1) following the previous study [23]. The PCR reaction mixture (25 μl) consisted 12.5 μl of 2× Red Dye Master mix (Ampliqon), 0.1 μM of each primer, 1 μl of DNA template, and molecular grade water. The PCR conditions were set as suggested by Bogaerts et al.*,* but was done as singleplex PCR with individual annealing temperatures as given in Table 1.

**Table 1:** Carbapenemases gene primers used for screening in the study. [23]^a^

Gene	Primer Sequence	Size (bp)	Annealing Temperature (°C)
*bla*_KPC-3_	F- TCGCCGTCTAGTTCTGCTGTCTTG	353	58.5
	R- ACAGCTCCGCCACCGTCAT		
*bla*_NDM-1_	F- ACTTGGCCTTGCTGTCCTT	603	51.9
	R - CATTAGCCGCTGCATTGAT		
*bla*_VIM_	F - TGTCCGTGATGGTGATGAGT	437	52.5
	R- ATTCAGCCAGATCGGCATC		
*bla*_IMP_	F- ACAYGGYTTRGTDGTKCTTG	387	51
	R- GGTTTAAYAAARCAACCACC		
*bla*_OXA-48_-like	F- ATGCGTGTATTAGCCTTATCG	265	52.5
	R- CATCCTTAACCACGCCCAAATC		

a Bp = basepairs; Wobble bases –Y(Pyrimidine),R(Purine),K (G/T),D (A/G/T); nucleotides – A,T,G,C.

### 2.7 Conjugation

Ten representative carbapenem-resistant isolates (positive for carbapenemase genes) were subjected to conjugation experiments based on a previously described study [24]. The recipient strain used was *E. coli* AB1157 (MTCC 1591) (strR). Donor and recipient cultures were grown overnight in LB broth supplemented with meropenem (4 μg/ml) and streptomycin (100 μg/ml), respectively, at 37 °C. Following incubation, the overnight cultures were then mixed in two different donor-to-recipient ratios (1:1 and 1:9) to facilitate conjugation using broth mating method. The mixtures were then incubated without shaking for 18 h at 37 °C. After incubation, 100 μl of the conjugation mixture was plated onto LB agar and Hichrome UTI agar supplemented with both meropenem and streptomycin to select the transconjugants. *E. coli* colonies were further subjected to genomic DNA extraction and screened for carbapenemase genes by PCR. To assess plasmid stability, the transconjugants were sub-cultured for three consecutive passages under non-selective conditions. Conjugation efficiency was calculated by using the formula: Conjugation efficiency = No. of Transconjugants (CFU/ml) / No. of donor cells (CFU/ml).

### 2.8 Whole genome sequencing and analysis

Five representative bacterial isolates were selected based on clinical relevance for WGS that includes *K. pneumoniae* (*n* *=* *2*), *Morganella morganii* (*n* *=* *2*), and *P. mirabilis* (*n* *=* 1). Genomic DNA from the isolates was extracted using Qia AMP Mini Kit (Qiagen, Germany). The extracted DNA was assessed for quality and quantified using the Qubit 4 Fluorometer (Invitrogen – Thermo Scientific, Waltham, MA, USA) and the SpectraMax QuickDrop UV–Vis Spectrophotometer (Molecular Devices, San Jose, California, USA). DNA libraries were prepared using the Nextera Library Preparation Kit (Illumina, San Diego, California, USA) and subjected to short-read sequencing using the Illumina Hiseq2000 platform, to produce 150 bp paired-end raw reads with 100× coverage. The raw data underwent quality check assessment using Fast (v.0.12.1). Raw reads were assembled using SPAdes v3.15.5 [25]. The quality of the assembled draft genome was evaluated using Quast v5.2.0 [26]. The draft genomes were annotated using Prokka v1.14.6 [27]. Antimicrobial resistance genes (ARGs) and virulence determinants were screened in the datasets using ABRicate v1.0.1 [28] with the Resfinder and CARD database for ARGs and the Virulence Factors Database for virulence factors (VFDB). Multi Locus Sequence typing was performed using pubMLST database [29] and MLST 2.0 [30]. ARGs, MLST and plasmid elements (with Plasmid Finder database) were analyzed using staramr v0.10.0 [31]. Mobile genetic elements were screened to detect associations of ARGs and virulence genes with mobile genetic elements using the MGE database (CGE server) available in the CGE server. Integron finder (v2.0.5) was used to screen for integrons [32]. Virulence factors were screened using Pathogen Finder [33].

## 3 Results

### 3.1 Carbapenem-resistant bacteria in chicken meat and MIC

**Fig. 2:**
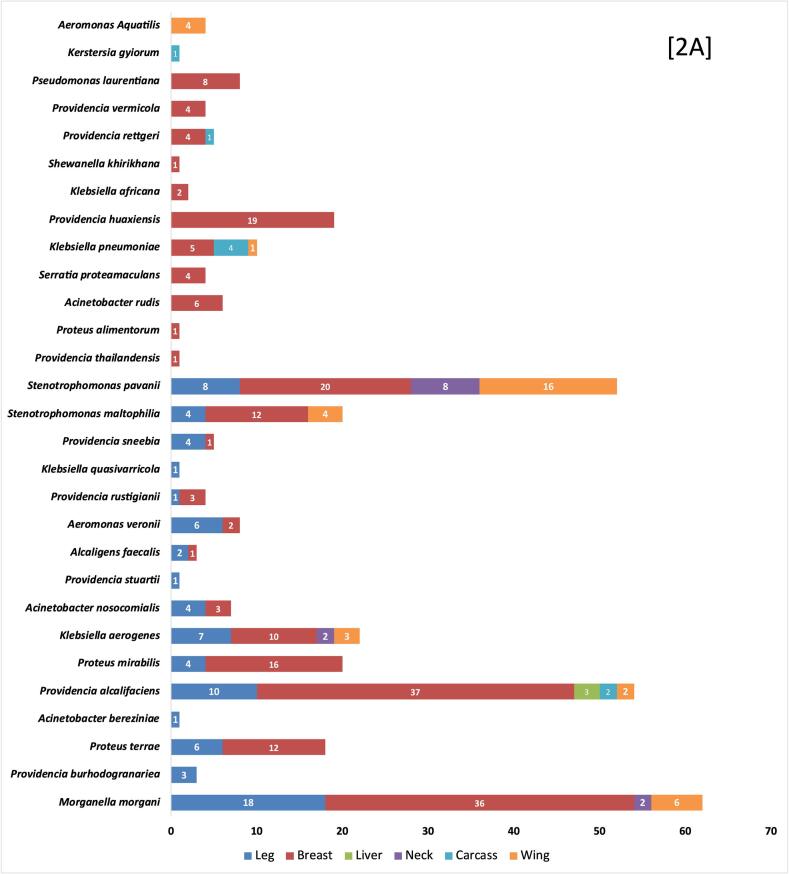

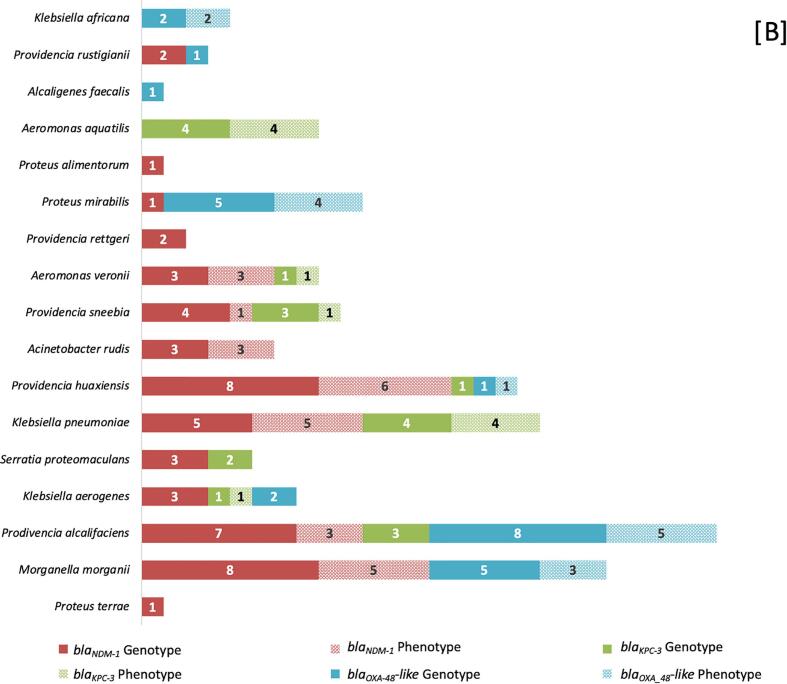
Distribution of Carbapenem-resistant bacteria among chicken meat samples. A- Prevalence of carbapenem-resistant bacteria species among different types of chicken meat samples. B- Distribution of carbapenemase genes and Phenotype (MHT-T: Modified Hodge test – TritonX-100) characteristics based on the bacterial species. Graphical representations were created using Microsoft Excel.

Chicken meat samples (*n* = 250) comprised of breast (*n* = 146), leg (*n* = 78), wings (*n* = 10) liver (*n* = 8), neck (*n* = 4), and carcass (*n* = 4). After pre-enrichment, 92 samples (36.8 %) showed growth in media supplemented with meropenem. After individually sub culturing colonies with different morphologies, a total of 368 isolates were obtained from 92 samples with an average of four isolates per sample. The isolates were identified by Sanger sequencing as follows: The most abundant species identified were *M. morganii (n* *=* 62*)*, *Providencia alcalifaciens (n* *=* 54*)*, *Stenotrophomonas pavanii (n* *=* 52*), Klebsiella aerogenes (n* *=* 22*)*, *Proteus mirabilis (n* *=* 20*), Stenotrophomonas maltophilia (n* *=* 20*), Providencia huaxiensis (n* *=* 19*)*, *Proteus terrae (n* *=* 18*)*, *K. pneumoniae (n* *=* 10*)* and the other species and their source are represented in Fig. 2A. During the identification process, 21 isolates could not be identified due to failure in reviving the isolates. These 21 isolates were excluded from the study and remaining 347 isolates were further processed. Minimum inhibitory concentrations of meropenem of the isolates were summarized in the Table 2. Out of 347 isolates, 73.4 % (255/347) were resistant to meropenem, 5.7 % (20/347) were intermediate resistant, and 20.7 % (72/347) were interpreted as intrinsic resistant since they belonged to *Stenotrophomonas* spp. Very high meropenem MICs (≥128 mg/l) were observed in *Aeromonas* spp., *Klebsiella* spp., *Proteus* spp. and *Providencia* spp.

**Table 2:** Minimum inhibitory concentration (MIC) of isolates obtained from the poultry source in Coimbatore, Tamil Nadu, India.

Species	Total no. of isolates	Minimum inhibitory concentration (mg/L) No. of isolates (% of isolates)^S/I/R^									
		≥128	≥64	≥32	≥16	≥8	≥4	≥2	≥1	≥0.5	≥0.25
*Acinetobacter bereziniae*	1	–	–	–	1(100) ^R^	–	–	–	–	–	–
*Acinetobacter nosocomialis*	7	–	–	2 (28.6) ^R^	3 (42.8) ^R^	2(28.6)^R^	–	–	–	–	–
*Acinetobacter rudis*	6	–	–	–	3 (50)^R^	3 (50)^R^	–	–	–	–	–
*Aeromonas aquatilis*	4	3 (75)^R^	1 (25)^R^	–	–	–	–	–	–	–	–
*Aeromonas veronii*	8	1 (12.5) ^R^	–	–	4 (50) ^R^	2 (25)^R^	1 (12.5)^I^	–	–	–	–
*Alcaligenes faecalis*	3	–	–	1 (33.3)^R^	2 (66.7)^R^	–			–	–	–
*Kersterisia gyiorum*	1	–	–	–	1 (100)^R^	–	–	–	–	–	–
*Klebsiella aerogenes*	22	–		2 (9.1)^R^	–	5 (22.72)^R^	15 (68.18) ^R^	–	–	–	–
*Klebsiella pneumoniae*	10	8 (80)^R^	1 (10)^R^	–	–	–	1 (10)^R^		–	–	–
*Klebsiella africana*	2	2 (100)^R^	–	–	–	–		–	–	–	–
*Klebsiella quasivarricola*	1	–	–	–	–	–	1 (100)^R^	–	–	–	–
*Morganella morganii*	62	–	3 (4.84)^R^	–	8 (12.9)^R^	17 (27.42) ^R^	23 (37.1) ^R^	11 (17.74)^I^	–	–	–
*Proteus alimentorum*	1	–	–	–	–	–	1(100) ^R^	–	–	–	–
*Proteus mirabilis*	20	1 (5)^R^	–	1 (5)^R^	–	11 (55) ^R^	2 (10) ^R^	5 (25)^I^	–	–	–
*Proteus terrae*	18	–	1 (5.55)^R^	5 (27.8)^R^	4 (22.22)^R^	–	8 (44.44) ^R^	–	–	–	–
*Providencia alcalifaciens*	54	3 (5.55)^R^	7 (12.96)^R^	4 (7.4)^R^	4 (7.4)^R^	19 (35.2) ^R^	15 (27.8) ^R^	2 (3.7)^I^	–	–	–
*Providencia burhodogrnariea*	3	–	–	–	–	–	3 (100)^R^	–	–	–	–
*Providencia huaxiensis*	19	–	–	–	3 (15.8)^R^	11 (57.9)^R^	5 (26.3)^R^	–	–	–	–
*Providencia rettgeri*	5	–	–	–	–	3 (60)^R^	2 (40)^R^	–	–	–	–
*Providencia rustigianii*	4	–	–	–	–	2 (50)^R^	1 (25)^R^	1 (25)^I^	–	–	–
*Providencia sneebia*	5	–	–	–	–	–	5 (100)^R^	–	–	–	–
*Providencia stuartii*	1	–	–	–	–	1 (100)^R^	–	–	–	–	–
*Providencia thailandensis*	1	–	–	–	–	–	1 (100)^R^	–	–	–	–
*Providencia vermicola*	4	–	–	–	–	–	4 (100)^R^	–	–	–	–
*Pseudomonas laurentiana*	8	–	–	–	–	8 (100)^R^	–	–	–	–	–
*Serratia proteamaculans*	4	–	–	–	–	–	4(100)^R^	–	–	–	–
*Shewanella khirikhana*	1	–	–	–	1(100)^R^	–	–	–	–	–	–
*Stenotrophomonas maltophilia*	16	–	–	–	4 (25)^NA^	4 (25) ^NA^	–	4 (25) ^NA^	4 (25) ^NA^	–	–
*Stenotrophomonas pavanii*	56	–	3 (5.35) ^NA^	9 (16.05) ^NA^	14 (25) ^NA^	8 (14.3) ^NA^	21(37.5) ^NA^	–	1 (1.8) ^NA^	–	–

The number of isolates including percentage with respect to each concentration of meropenem was described. MIC of meropenem values were interpreted using CLSI guidelines M100 - Performance Standards for Antimicrobial Susceptibility Testing, 34th edition, 2024. * S-susceptible; I- intermediate resistance; R – resistance, NA- not applicable.

### 3.2 Carbapenemase genes dissemination

Totally, 95 among 347 isolates were identified with carbapenemase genes. *bla*_NDM-1_ was most common 14.7 % (51/347), detected mainly in *M. morganii (n* *=* *8)* and *P. huaxiensis (n* *=* *8)* followed by *P. alcalifaciens (n* *=* *7), K. pneumoniae (n* *=* *5)*, *Providencia sneebia (n* *=* *4)*, *K. aerogenes (n* *=* *3)*, *Serratia proteomaculans (n* *=* *3)*, *Acinetobacter rudis (n* *=* *3)*, *Aeromonas veronii (n* *=* *3)*, *Providenica rettgeri (n* *=* *2), Providencia rustigianii (n* *=* *2)*, *P. terrae (n* *=* *1)*, *Proteus alimentorum (n* *=* *1)* and *P. mirabilis (n* *=* *1)* (Fig. 2B).The second-most frequently detected carbapenemase type was *bla*_OXA-48_-like*,* detected in 25 isolates 7.2 % (25/347). Carbapenemases of the *bla*_OXA-48-_like subtype was highly detected in *P. alcalifaciens (n* *=* *8)* followed by *P. mirabilis (n* *=* *5)*, *M. morganii (n* *=* *5)*, *K. aerogenes (n* *=* *2)*, *Klebsiella africana (n* *=* *2), A. faecalis (n* *=* *1)*, *P. rustigianii (n* *=* *1)* and *P. huaxiensis (n* *=* *1)*. Lastly, *bla*_KPC-3_ was detected in 5.4 % (19/347). Highest number of *bla*_KPC-3_ were detected in *K. pneumoniae* (*n* = 4) and *Aeromonas aquatilis* (*n* = 4), followed by *P. sneebia* (*n* = 3), *P. alcalifaciens* (*n* = 3), *P. huaxiensis* (*n* = 1), *S. proteamaculans* (*n* = 2), *K. aerogenes* (*n* = 1) and *A. veronii* (*n* = 1) (Fig. 2B). No isolates tested positive for *bla*_VIM_ and *bla*_IMP_. Multiple resistance genes were identified among individual isolates. One *P. alcalifaciens* and one *P. huaxiensis* were found to co-produce *bla*_NDM-1_ and *bla*_OXA-48_-like. Similarly, two *P. sneebia*, one *P. alcalifaciens,* and one *S. proteomaculans* were co-producing *bla*_NDM-1_ and *bla*_KPC-3_.

### 3.3 Carbapenamase phenotype and trans-conjugation

Among the95 carbapenemase gene-positive isolates, 54 isolates were identified to produce carbapenemases in the MHT (Fig. 2B). Highest number of carbapenemases genes were detected in *P. alcalifaciens*(8/18) isolates being carbapenemase producing phenotype in MHT, followed by *M. morganii* (8/13), *P. huaxiensis* (7/10), *K. pneumoniae* (9/9), *P. sneebia* (2/7), *K. aerogenes* (1/6), *P. mirabilis* (4/6),*A. veronii* (4/4), *A. aquatilis* (4/4), *A. rudis* (3/3) and *K. africana* (2/2). Although few species were carrying carbapenemase genes, they were not observed for carbapenemase production that includes *P. terrae* (0/1), *A. faecalis* (0/1), *S. proteomaculans* (0/5), *P. rustigianii* (0/3*), P. rettgeri* (0/2) and *P. alimentorum* (0/1).

Two isolates carrying both *bla*_NDM-1_ and *bla*_OXA-48_-like genes were positive in the modified hodge test. One *P. sneebia* isolate co-producing *bla*_NDM-1_ and *bla*_KPC-3_ was identified positive in MHT. Nine isolates were found to be carbapenemase producers though they were negative for carbapenemase genes. MHT plate images were depicted in Supplementary Fig. 2. A total of 10 isolates were subjected to conjugation experiments including eight isolates that were *bla*_NDM-1_ positive including *P. alcalifaciens* (*n* = 3), *K. aerogenes* (*n* = 1), *K. pneumoniae* (*n* = 1), *M. morganii* (*n* = 1) and *P. mirabiliis* (*n* = 1)*,* while one was *bla*_KPC-3_ positive (*K. pneumoniae*) and two *bla*_OXA-48_-like positive *M. morganii* isolates. Out of 10, one *K. pneumoniae* (*bla*_NDM-1_), one *M. morganii* (*bla*_NDM-1_) and one *P. alcalifaciens* (*bla*_NDM-1_) had successfully facilitated inter-generic transfer of the ARGs into the recipient cell. Conjugation efficiency in CFU/ml of *K. pneumoniae, M. morganii* and *P. alcalifaciens* were 1.1 × 10^−8^, 5.3 × 10^−9^ and 7.1 × 10^−9^ respectively.

### 3.4 Genome characteristics of whole genome sequenced isolates

Five isolates were selected for sequencing, comprising two *K. pneumoniae*, two *M. morganii* and one *P. mirabilis*. The length of raw reads was approximately ranging from ∼150 bp. The mean Phred scores for all datasets ranged from 32 to 33. *K. pneumoniae* genome sizes were approximately 5.6 and 5.7 Mbp, while *M. morganii* genome sizes were 3.8 and 3.9 Mbp. *P. mirabilis* genome size was 3.9 Mbp. Two *K. pneumoniae* were identified as MLST sequence type ST15 and ST16. *P. mirabilis* belonged to ST135. MLST typing could not be performed for both *M. morganii a* and b isolates, due to the absence of available typing scheme for this species MLST tools at the time of analysis.

### 3.5 ARGs, Virulence factors and mobile genetic elem*ent*s

The ARGs detected in *K. pneumoniae* ST15 and ST16 demonstrated extensive multi-drug resistance mechanisms (Table 3). Virulence determinants were exclusively identified only in the *K. pneumoniae* isolates. Other species produced no hit during VFDB analysis. Virulence factors of *K. pneumoniae* included genes associated with outer-membrane protein A (*ompA*), adherence, biofilm formation (*fyuA* and *yag/ecp complexes*), siderophore production (*ent, irp* and *ybt*) and transporter protein (*fepC*, Table 3). Mobile genetic elements such as plasmids, insertion sequences, integrons and transposons carried by the WGS isolates are listed in Table 3. Mobile genetic elements and their association with ARGs and virulence genes were studies as follows: *qnrD1* was identified in the *Col3M* plasmid of *M. morganii* (isolate a). In *K. pneumoniae*, ST16, *bla*_OXA-181_ and *qnrS1* were associated with the insertion sequence *ISKpn19* in the *ColKP3* plasmid, while virulence genes such as *fyuA* and *irp2* were present on a contig containing two *ISEc36* and *ISEc33* insertion sequences. Additionally, *mph(A)* was associated with insertion sequence IS6100 in a contig. In *P. mirabilis,* aminoglycoside resistance genes (*AAC(3)-Iid*, *APH(6)-Id* and *APH(3″)-Ib*) were detected on the *IncQ1* plasmid and *qnrD1* on the *Col3M* plasmid. Integrons including class one integrons (*intI*) and *attC* were present in one or more contigs of *K. pneumoniae* ST16 and *P. mirabilis.* The pathogenic nature of the isolates was estimated by the probability score in PathogenFinder, where *Proteus mirabilis* ST135 (probability score – 0.778)*, K. pneumoniae* ST15 (probability score − 0.88), *K. pneumoniae* ST16 (probability score − 0.874), and *M. morganii* (a) (probability score – 0.65) were predicted as pathogenic to human; whereas *M. morganii* (b) was classified as non-pathogenic in human (probability score 0.502) using the PathogenFinder tool, though it is an opportunistic pathogen.

**Table 3:** Genome characteristics of whole genome sequenced chicken meat isolates.

Isolate (Total genome length in bp)	MLST	ARGs using CARD database	Virulence genes	Plasmid Finder	Mobile genetic elements and integrons
*Klebsiella pneumoniae* (5,642,775)	ST15	*AAC(3)-Iie, bla*_OXA-1_*, AAC(6′)-Ib-cr,* *OmpK37, catII, bla*_TEM-1_*, ramA, marA, cpxA, KpnH, KpnG, emrR, baeR, mdtC, mdtB, acrD, oqxA, oqxB,H-NS, FosA6, msbA, KpnE, KpnF, acrA, acrB, mphA, QnrB17, ANT(3″)-IIa, tet(A), bla*_SHV-134_*, CRP*	*yagV/ecpE, yagW/ecpD,* *yagX/ecpC, yagY/ecpB, yagZ/ecpA, ykgK/ecpR, ompA, fepC, entB, entA*	*ColpVC,* *IncFIB(K), IncFIB(pKPHS1), IncFIB(pQil), IncN*	*ISVsa3, IS6100, IS903, ISApuI, ISKpn28, Tn5403, ISKpn21, ISEclI, ISKpn26, ISKpn14*
*Morganella morganii (a)* (3,908,411)	–	*tet(B), QnrD1, bla*_DHA-13_*, CRP*	*–*	*Col3M*	*–*
*Klebsiella pneumoniae* (5,724,861)	ST16	*dfrA14, bla*_SHV-1_*, marA, msbA,* *KpnE, KpnF, CRP, cpxA, FosA5, oqxA, oqxB, emrR, KpnG, KpnH, ramA, OmpK37, acrD, bla*_OXA-181_*, QnrS1, bla*_NDM-5_*, determinant_of_bleomycin_resistance, sul1, aadA2, dfrA12, bla*_TEM-1_*, rmtB,* bla_CTX-M-15_*, mphA, H-NS, acrB, acrA, baeR, mdtC, mdtB*	*fepC, entB, entA, fyuA, ybtE, ybtT,* *ybtU, irp1, irp2, ybtA, ybtP, ybtQ, ybtX, ybtS, ompA, ykgK/ecpR, yagZ/ecpA, yagY/ecpB, yagX/ecpC, yagW/ecpD, yagV/ecpE*	*ColKP3, Col440II, Col440I, FIA(pBK30683), IncFIB(K), IncFII(K), IncX3*	*ISKpn19, ISEc36, ISEc33, ISKpn14, ISKpn33, ISEclI, ISKox3, IS6100,IntI, attC*
*Morganella morganii (b)* (3,880,930)	–	*bla*_DHA-5_*, CRP*	*–*	*Col3M*	*Col3M, IS3, IS51*
*Proteus mirabilis* (3,962,569)	ST135	*catA4, dfrA1, SAT-1, ANT(3″)-Iia,* *AAC(3)-Iid, APH(6)-Id, APH(3″)-Ib, sul2, sul1, aadA5, dfrA17, catI, QnrD1, bla*_TEM-1_*, tet(J), APH(3′)-Ia, CRP*	*–*	*Col3M, IncQ1*	*ISVsa5, Tn7, IS629, IS5, IS26,IntI, attC*

The genome of sequenced isolates was categorized based on MLST, antimicrobial resistance determinants, virulence factors and mobile genetic elements such as plasmid elements and insertion elements. Insertion elements*CDS – Coding sequences; ARGs – Antimicrobial resistance genes; MLST- Multi Locus Sequence typing.

## 4 Discussion

In a study conducted in Tamil Nadu, the prevalence of carbapenem-resistant bacteria in clinical samples was reported to be 71 % and 68 % among 151 isolates for meropenem and imipenem, respectively [24]. While the overall burden of carbapenem-resistant bacteria in India is well-documented [34], our study highlighted their presence in raw chicken meat samples, a source that remains underexplored. This finding contributes valuable insight into the potential reservoirs and dissemination pathways of ARGs to clinically significant bacteria. In our study, we detected carbapenem-resistant bacteria in 36.8 % (92/250) of chicken meat samples. Su et al. reported prevalence of broiler origin carbapenem-resistant *E. coli*, *P. mirabilis* and *K. pneumoniae* isolates to be 3.57 % (6/168), 10 % (5/50) and 3.03 % (1/33), respectively in China [35]. All of these isolates were carrying both *bla*_NDM-1_ and *bla*_OXA-48_-like [35]. However, in our study, *E. coli* or *Salmonella* spp. was not identified which is noteworthy. In our study, we observed few multiple genotype positive isolates but only one *P. sneebia* (*bla*_NDM-1_+ *bla*_KPC-3_) was producing carbapenemase. We speculate that other resistance mechanisms like efflux pumps and porins might play role even though harbouring gene. In a study conducted in Egypt, the study authors reported that 42 % (15/35) isolates were carbapenem-resistant and most common types described were *bla*_KPC-3_*, bla*_NDM-1_ and *bla*_OXA_*.* In the same study, 11 isolates were co-harbouring *bla*_KPC-3_*, bla*_NDM-1_ and *bla*_OXA-48_-like, three isolates were carrying both *bla*_KPC-3_ and *bla*_NDM-1_ and a isolate co-harbouring *bla*_OXA-48_-like and *bla*_NDM-1_ [36]. Recently, it was discovered that *bla*_NDM-1_ and *bla*_OXA-48_-like carbapenemases together increases virulence fitness when conjugated into bacteria [37]. Furthermore, carrying multiple carbapenemase genotype may influence the degree of resistance to carbapenems [38]. Other studies from India reported the absence of *bla*_VIM_ and *bla*_IMP_ [34]. Consistently, in our study we did not detect *bla*_VIM_ and *bla*_IMP_. Furthermore, both *K. pneumoniae* ST15 and ST16 were subjected to conjugation studies, where one *K. pneumoniae* ST16 transconjugant was obtained among three transconjugants.

*K. pneumoniae* ST15 is considered as a common lineage in poultry sectors [39,40]. The study also described the poultry-origin *K. pneumoniae* ST15 loaded with ARGs showed genetic relatedness to human clinical strains [40]. Recently, carbapenem-resistant *K. pneumoniae* ST15 clones were classified as high-risk clones in China [41]. Further, other β-lactamases found in *K. pneumoniae* ST15 in this study were *bla*_OXA-1_ and bla_SHV-134_. While ST16 is more frequently recognized as the international high-risk clone isolated from clinical settings rather than animal origin [42,43]. In our study, poultry origin *K. pneumoniae* ST16 identified was co-harbouring *bla*_SHV-1_*, bla*_NDM-5_*, bla*_OXA-181_ and *bla*_CTX-M-15_, which are more ARGs than the clinical origin strains from Italy [42] and Iran [43]. In addition, both ST15 and ST16 genomes in our study was packed with efflux pump-based variants of ARGs such as *CRP*, *mdt*, *acr*, *oqx*, *bae*, *emr*, *Kpn*, *cpx* and *mar* genes. Lastly, carbapenemase-producing *P. mirabilis* ST135 was reported only in clinical samples from Germany unlike our study [44].

Virulence factors id*ent*ified in our study are pivotal in host pathogenesis, for example, the *yag/ecp* and *ykg/ecp* complexes facilitate cell adherence during infection, while siderophores such as *fep, ent, fyu, ybt* and *irp* type siderophores are involved in iron sequestration.

Mobile genetic elements such as plasmid elements were identified across all datasets. Similar to our observations, *ColpVC, IncFIB(K), IncFIB(pKPHS1), IncFIB(pQil), IncN* have been previously associated with the ST15 lineage of *K. pneumoniae* [45]. Two separate studies also reported described the presence of *ColKP3*, *Col440II*, *Col440I*, *FIA (pBK30683)*, *IncFIB(K)*, *IncFII(K)* and *IncX3* plasmids in *K. pneumoniae* ST16, consistent with our findings [42,46]. *P. mirabilis* ST135 in our study carried the *Col3M* and *IncQ1* plasmids, which is consistent with a previous study [47]. Furthermore, the genomic maps of *K. pneumoniae* ST15 and ST16, along with ARGs and virulence factors, are represented in Supplementary Fig. 1. The transmission of such pathogens between humans, poultry, and the environment has been discussed in several studies [48,49]. We speculate that these internationally-disseminated clones may have been introduced through farming personnel, contaminated with poultry feed, or exposure to polluted water sources. In an another study, different strains of poultry-origin and human-origin *P. mirabilis* isolates exhibited similar antibiotic resistance pattern, highlighting the potential risks associated with poultry products in human infections [50]. In 2016, a study in Portugal reported a multidrug-resistant, avian origin *M. morganii* carrying *qnrD1* within a *Col3M* plasmid similar to the *M. morganii* (a) in our study [51]. Furthermore, based on PathogenFinder results, four of the isolates have a high probability of being human pathogens.

The limitations of the study were that not all the isolates were sequenced due to the financial limitations, sequencing all the isolates might have given detailed insights on the mechanism and clonal lineage-based prevalence in chicken meat samples. That underpins the necessity for more studies on the genome characteristics of pathogens such as *Proteus* spp. and *Morganella* spp. to avail more data. In addition, few of the isolates screened for carbapenemase genes were not revived for species identification. Several isolates were identified as *Stenotrophomonas* spp., a known nosocomial pathogen that is intrinsically resistant to carbapenems. Previous studies, have reported occasional colonization of this organism in food products such as. chicken eggs, fish and salads [[52], [53], [54]]. Despite its intrinsic resistance, *Stenotrophomonas* spp., in our study exhibited varied MIC values for carbapenems. Interestingly, some isolates lacking carbapenemase encoding genes, still demonstrated phenotype carbapenemase activity. This may be due to the fact that we screened only for the most commonly occurring carbapenemase genes, and did not include less frequent variants such as such as *bla*_GES_*, bla*_DIM_*.* Furthermore, some retail shops refused sampling, thus limiting the diversity of samples from the district. According to the previous reports, we speculate that misuse and overuse of antibiotics without proper antibiotic stewardship in LMICs might have led to the transmission of drug-resistant pathogens to unforeseen sources like demonstrated in this study. Nevertheless, antibiotic usage for poultry industries is anticipated to increase by 82 % by 2030 in India [55].

In our study, we identified carbapenem-resistant bacteria in the raw chicken meat samples of poultry farm origin, where carbapenems are not generally used. However, antibiotics such as oxytetracycline, tetracycline, chlortetracycline, doxycycline enrofloxacin, ciprofloxacin, colistin and neomycin are widely used in the Indian poultry industry. Notably, some of the clinically significant bacteria identified in our study including *Proteus* spp., *Klebsiella* spp., *Pseudomonas* spp. and *Serratia* spp. Furthermore, the study was conducted in a region, recognized as a major poultry hub in India, one of the largest poultry-producing states in the country.

## 5 Conclusion

The current study investigated the prevalence of carbapenem-resistant bacteria in chicken meat samples from one of the largest global producers of poultry. In our study, we found similar species of CRB from chicken meat to the reports from other countries. WGS revealed unusual Sequence types of *K. pneumoniae* (ST16) in the poultry source, which is limited to clinical setting and are harbors a greater number of β- lactamase genes compared to the one originated from clinical setting. This suggests the importance of surveillances of ARGs in food animal sources especially in unexplored LMICs that lack meticulous regulations on the usage of antimicrobials for animal food production. This study described the detailed insights on the different types of species carbapenemase producing bacteria in poultry sources in India although the carbapenems have not been used for poultry farming. Thus, this study will widen the knowledge on the lacking details regarding the ARGs in the food chicken in the country and foster the antimicrobial stewardship in poultry sectors.

## Funding statement

A PSG Institutions Management Grant supported this study.

## CRediT authorship contribution statement

**Seshan Sivasankar:** Methodology, Formal analysis, Writing – original draft, Writing – review & editing. **Martin Peter Grobusch:** Methodology, Writing – review & editing. **Frieder Schaumburg:** Writing – review & editing. **Sankarganesh Jeyaraj:** Conceptualization, Methodology, Formal analysis, Writing – review & editing.

## Declaration of competing interest

The authors declare that they have no known competing financial interests or personal relationships that could have appeared to influence the work reported in this paper.

## Data Availability

Data will be made available on request.

## Appendix A Supplementary data

Supplementary material Image 1
