# Supplementary material for: Prevalence and characterization of carbapenem-resistant gram-negative bacteria from poultry in Tamil Nadu, India
Source: One Health. 2025 Sep 5;21:101192. doi: 10.1016/j.onehlt.2025.101192 (PMC12696407; doi:10.1016/j.onehlt.2025.101192)
Supplement: Supplementary file 1 — Supplementary material [file mmc1.docx]

**Supplementary Figure 1.** Genomic map of ST15 and ST16 isolates of K. pneumoniae.


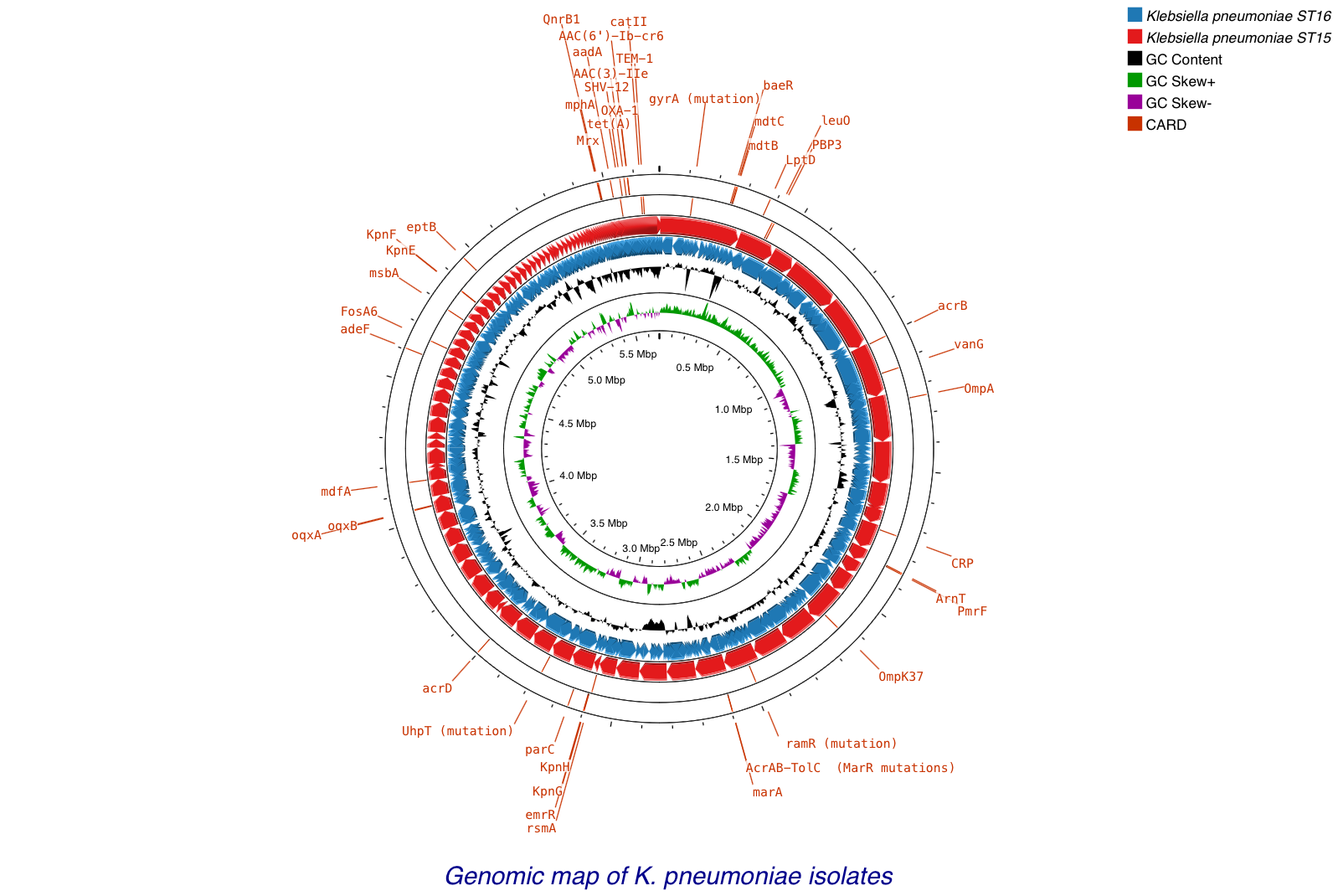


Supplementary Figure 1: Genomic maps of *K. pneumoniae* ST15 and ST16 isolates constructed in Proksee.

**Supplementary Figure 2.** Representative images of Modified Hodge test results.


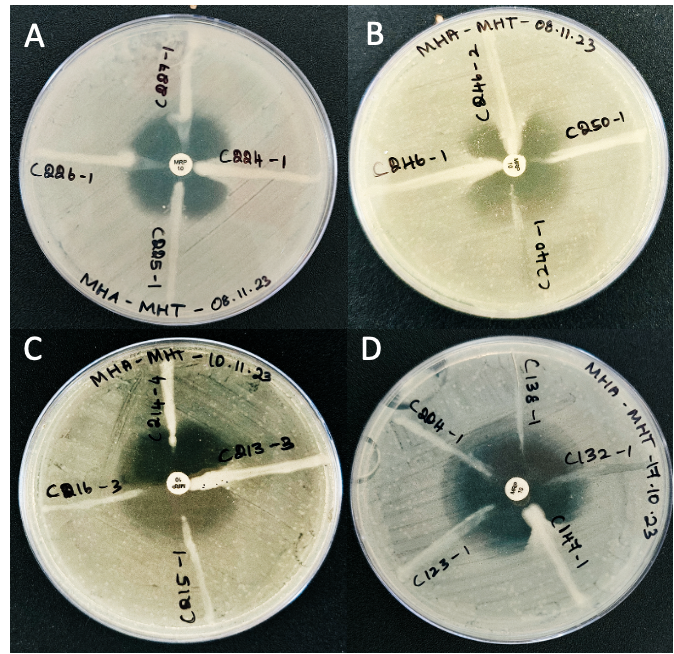


Supplementary Figure 2: **A**- K. pneumoniae ST16 (C224-1) was positive in MHT. **B** – P. mirabilis (C246) was positive. **C** – K. pneumoniae ST15. **D** – A. aquatilis (C147-1).
